# Tick-Borne Encephalitis Virus, United Kingdom

**DOI:** 10.3201/eid2601.191085

**Published:** 2020-01

**Authors:** Maya Holding, Stuart D. Dowall, Jolyon M. Medlock, Daniel P. Carter, Steven T. Pullan, James Lewis, Richard Vipond, Mara S. Rocchi, Matthew Baylis, Roger Hewson

**Affiliations:** National Institute for Health Research Health Protection Research Unit in Emerging and Zoonotic Infections, Liverpool, UK (M. Holding, S.D. Dowall, J.M. Medlock, D.P. Carter, R. Vipond, M.S. Rocchi, M. Baylis, R. Hewson);; Virology and Pathogenesis Group, National Infection Service, Public Health England, Porton Down, UK (M. Holding, S.D. Dowall, R. Vipond, R. Hewson);; Medical Entomology and Zoonoses Ecology, Emergency Response Department, Public Health England, Porton Down (M. Holding, J.M. Medlock);; Genomics, National Infection Service, Public Health England, Porton Down (D.P. Carter, S.T. Pullan);; Geographic Information Systems, Emergency Response Department, Public Health England, Porton Down (J. Lewis);; Virus Surveillance Unit, Moredun Research Institute, Edinburgh, Scotland, UK (M.S. Rocchi);; Institute of Infection and Global Health, University of Liverpool, Liverpool (M. Baylis)

**Keywords:** meningitis/encephalitis, viruses, tick-borne encephalitis, tick-borne encephalitis virus, TBEV, louping ill virus, sentinel animals, deer, United Kingdom, immunological surveillance, tickborne infections, Ixodes ricinus, zoonoses, ticks, flavivirus, vector-borne infections

## Abstract

During February 2018–January 2019, we conducted large-scale surveillance for the presence and prevalence of tick-borne encephalitis virus (TBEV) and louping ill virus (LIV) in sentinel animals and ticks in the United Kingdom. Serum was collected from 1,309 deer culled across England and Scotland. Overall, 4% of samples were ELISA-positive for the TBEV serocomplex. A focus in the Thetford Forest area had the highest proportion (47.7%) of seropositive samples. Ticks collected from culled deer within seropositive regions were tested for viral RNA; 5 of 2,041 ticks tested positive by LIV/TBEV real-time reverse transcription PCR, all from within the Thetford Forest area. From 1 tick, we identified a full-length genomic sequence of TBEV. Thus, using deer as sentinels revealed a potential TBEV focus in the United Kingdom. This detection of TBEV genomic sequence in UK ticks has important public health implications, especially for undiagnosed encephalitis.

The only tickborne flavivirus in the United Kingdom documented to cause disease in vertebrates is louping ill virus (LIV), a vivirus transmitted by the deer/sheep tick, *Ixodes ricinus* (*1*). This tick species is the most abundant and widely distributed tick species in the United Kingdom and a known vector of Lyme borreliosis. LIV is most commonly detected in sheep, cattle, and red grouse and has been reported in Scotland, Wales, and England (primarily Cumbria, Devon, and North Yorkshire) (*1*). Humans are incidental hosts for LIV, and infection has been reported infrequently; ≈45 clinical cases have been linked to encephalitis during the past 85 years (*1*,*2*). However, the short window of acute infection leads to uncertainty about whether suspected cases resulted from LIV infection or some other cause, although serologic analysis to analyze recent exposure through induction of IgM-specific responses, in combination with clinical symptoms, could inform a presumptive diagnosis. Human cases are mostly linked to occupational exposure, particularly in abattoir or farm workers and occasionally in laboratory staff (*2*). Although the UK Animal and Plant Health Agency holds a database of confirmed diagnoses of LIV in livestock (*3*,*4*), the distribution and regional prevalence of LIV has not been fully defined. Records of distribution and regional prevalence are based on voluntary submissions by farmers and veterinarians from symptomatic livestock (*1*), from which private submissions are not integrated. Serologic analysis has been complicated; some animals received vaccination before its withdrawal.

Tick-borne encephalitis virus (TBEV) is a closely related flavivirus that, although known to be less virulent than LIV for sheep (*5*), causes a neurologic disease (tick-borne encephalitis [TBE]) after transmission to humans by infected ticks, producing clinical disease in an estimated one third of TBEV infections (*6*). TBE typically has a biphasic course starting with a prodromal phase with influenza-like symptoms, followed by a symptom-free interval before neurologic disease occurs; neurologic disease ranges from mild meningitis to severe encephalitis with or without myelitis and spinal paralysis (*7*). Three classic subtypes of TBEV are recognized: European (TBEV-Eu), Siberian, and Far Eastern. Two additional TBEV subtypes have recently been proposed: Baikalian subtype and the Himalayan subtype (*8*). TBEV-Eu is the prevailing subtype in Western Europe where it is primarily transmitted by *I. ricinus* ticks and is maintained within forest and meadow biotypes in endemic foci. In the United Kingdom, TBE is considered an imported disease; opportunities for the virus to become established principally are limited because the UK climate was not thought to support the specific conditions required for enzoonotic cycles to be established for TBEV to become endemic (*9*). However, changes in climate have affected the emergence, distribution, and abundance of *I. ricinus* in the United Kingdom (*10*); thus, the risk for tickborne disease has increased (*11*). A recent study provided evidence that co-infestation of tick larvae and nymphs occurs in small mammals in UK woodland (*12*). The increasing range of TBEV in Western Europe was underscored recently when the Netherlands reported its first human case in 2016 (*13*). Moreover, retrospective serologic screening of deer serum samples and molecular analysis of questing ticks found evidence of TBEV circulation in the Netherlands as far back as 2010 and 2015 (*13*,*14*). Given the increasing possibility that TBEV could be circulating in the United Kingdom, Public Health England developed a surveillance program focusing on wild animals and ticks.

In TBEV-endemic areas in continental Europe, the prevalence of TBEV in questing ticks is low, rarely exceeding 1% even in regions where the incidence of human infections is high (*15*). Therefore, instead of screening ticks directly, we used sentinel animals first to identify serologic evidence of TBEV to highlight sites for focused tick testing by specific TBEV detection using real-time reverse transcription PCR (rRT-PCR). Deer are proven as reliable sentinels for identifying areas where TBEV is present (*13*,*15*) because they have a limited home range, are available in large numbers, and are broadly dispersed within the surveillance areas. They also show long-lasting antibody responses after natural exposure to flaviviruses (*15*,*16*).

For our study, collectors retrieved blood samples from deer culled in England and Scotland during February 2018–January 2019; when available, they also collected tick samples. We tested the blood samples for TBEV or LIV antibodies and the ticks for the presence of viral RNA by rRT-PCR. 

## Methods

### Sample Collection

We recruited persons involved in routine management of deer from across the United Kingdom to collect serum and tick samples from any species of deer. This program was promoted through organizations involved in deer management. These deerstalkers submitted 1,323 serum samples (and tick samples where present) from deer culled in England and Scotland during February 2018–January 2019. The University of Liverpool Ethics Committee (ref: VREC596) granted ethics approval for this study on February 1, 2018.

Blood samples were collected in serum-separation vacutainers from the chest cavity during gralloching, and blood-fed ticks were collected from any location on the deer carcass. Samples were centrifuged at 1,500 relative centrifugal force for 10 min and aliquoted. Serum and tick samples were stored at −80°C until further processing.

### ELISA Testing

We tested serum samples for antibodies to TBEV using the commercial Immunozym FSME IgG All Species ELISA (Progen, https://www.progen.com) according to the manufacturer’s instructions. We read plates at an optical density ratio of 450 nm. We considered samples with a reading of >127 Vienna units/mL to be seropositive.

### Hemagglutination Inhibition Testing

We tested serum samples for antibodies to LIV using a hemagglutination inhibition (HAI) test (*17*,*18*). We considered samples with a titer >20 seropositive. A small number of samples did not have sufficient serum for HAI testing.

### Tick Identification and RNA Extraction

We morphologically identified all ticks collected from culled deer within a 15-km radius of any TBEV ELISA–seropositive deer (*19*) to life stage and species level. We individually homogenized the ticks in 300 μL RLT buffer (QIAGEN, https://www.qiagen.com) in MK28-R Precellys homogenizing tubes using a Precellys 24 homogenizer (Bertin, https://www.bertin-instruments.com) at 5,500 rpm for 5 sec, followed by a 30-sec break; we repeated this process 4 times. We then added 300 μL of isopropanol and passed the tick homogenate through a QIAshredder (QIAGEN). We extracted total RNA using the BioSprint 96 One-For-All Vet Kit (QIAGEN) and eluted it into 100 μL AVE buffer according to the manufacturer’s instructions.

### rRT-PCR

We tested individual tick samples for LIV/TBEV RNA using a sensitive LIV/TBEV assay (*20*). We amplified RNA in 20 μL rRT-PCR mix containing 0.8 μL Invitrogen (https://www.thermofisher.com) Superscript III/Platinum Taq Mix, 10 μL Invitrogen 2X reaction mix, 1.6 μL 50 mmol/L MgSO_4,_, 1 μL of 1 μmol/L forward primer, 1 μL of 18 μmol/L reverse primer, 0.2 μL of 25 μmol/L probe, 5 μL template, and 0.4 μL molecular-grade water. 

We also tested all RNA-positive samples using a secondary assay designed to detect only LIV (*21*). We amplified RNA in 20 μL rRT-PCR mix containing 0.8 μL Invitrogen Superscript III/Platinum Taq Mix, 10 μL Invitrogen 2X reaction mix, 0.8 μL of 10 μmol/L forward primer, 1.8 μL of 10 μmol/L reverse primer, 1.0 μL of 5 μmol/L probe, 5 μL template, and 0.6 μL molecular-grade water.

### Sequencing and Phylogenetic Analysis

We prepared the tick sample that showed a high level of TBEV RNA for metagenomic RNA sequencing (*22*) and assembled the sequencing data using SPAdes version 3.1.1 (*23*). We inferred the evolutionary history by using the maximum-likelihood method based on the Tamura 3-parameter model (*24*). We used the tree with the highest log likelihood. We automatically obtained initial trees for the heuristic search by applying neighbor-joining and BioNJ (*25*) algorithms to a matrix of pairwise distances estimated using the maximum composite likelihood approach and then selecting the topology with superior log likelihood value. The analysis involved 10 full-length genomic TBEV nucleotide sequences and was performed using Molecular Evolutionary Genetics Analysis version 7.0 software (*26*).

## Results

**Figure 1:**
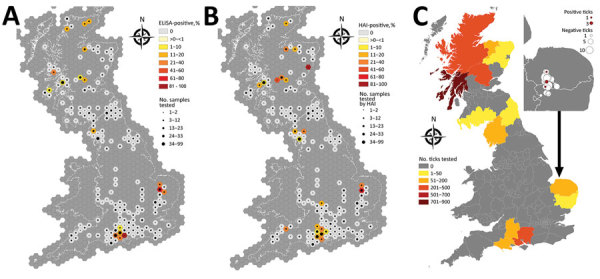
Results for deer serum samples and ticks tested for tick-borne encephalitis virus, United Kingdom. A, B) Number of samples tested and seroprevalence of samples positive by ELISA (A) and HAI (B). C) Number of ticks tested by county; inset shows magnification of testing area with ticks positive by real-time reverse transcription PCR. HAI, hemagglutination inhibition. Source: Ordnance Survey data, © Crown copyright and database right 2019; and National Statistics data, © Crown copyright and database right 2019.

Deerstalkers submitted a total of 1,323 serum samples, of which 14 samples were excluded from analysis because of insufficient location or deer species information. Serum samples were obtained from 5 deer species and a hybrid of 2 species; 61% of samples submitted were from male deer. The most frequently sampled species were roe deer (*Capreolus capreolus*) (51%), followed by fallow deer (*Dama dama*) (19%). Samples were submitted from across Scotland and England, but distribution and density of samples varied by county (Figure 1, panel C). A limited number of samples were submitted from across the Midlands and parts of Northern England; no samples were submitted from Wales.

Of serum samples from across the United Kingdom, 4% were positive by ELISA, and 5% by HAI. Cohen’s κ indicated substantial agreement (0.61) between the methods, indicating ELISA results agreed closely with HAI test results (Table 1). ELISA yielded positive results in all deer species for which it was used. These were 27/663 roe, 10/246 fallow, 9/242 red deer (*Cervus elaphus*), 6/108 muntjac (*Muntiacus reevesi*), 1/48 sika (*Cervus nippon*), and 0/2 red/sika hybrids. HAI determined the following positives: 28/662 roe, 15/245 fallow, 18/242 red, 7/106 muntjac, 0/45 sika, and 1/2 red/sika hybrid.

**Table 1:** Variation between ELISA for tick-borne encephalitis virus and HAI for louping ill virus, United Kingdom

ELISA result	HAI result		Not tested	Total
	Positive	Negative†		
Positive	38	14	1	53
Negative‡	31	1,219	6	1,255
Total	69	1,233	7	1,309

*HAI, hemagglutination inhibition. †HAI negative, borderline, unknown. ‡ELISA negative/borderline.

ELISA- and HAI-positive samples were geographically distributed to specific areas (Figure 1, panels A, B); seroprevalence was high in southwestern Norfolk and northwestern Suffolk (Thetford Forest) in the east of England. Norfolk had the highest seroprevalence detected by ELISA (51.4%), followed by Hampshire (14.3%), Suffolk (10.7%), and Scottish Highlands (8.6%) (Table 2).

**Table 2:** ELISA- and HAI-positive results for tick-borne encephalitis virus from counties from which serum samples were submitted, United Kingdom*

County and country	ELISA			HAI	
	No. positive/no. tested	% Positive (95% CI)†		No. positive/no. tested	% Positive (95% CI)†
Norfolk, England	18/35	51.43 (35.57–67.01)		16/35	45.71 (30.46–61.82)
Hampshire, England	15/105	14.29 (8.74–22.35)		14/104	13.46 (8.07–21.46)
Suffolk, England	3/28	10.71 (2.90–28.01)		2/28	7.14 (0.90–23.73)
Highland, Scotland	7/81	8.64 (3.99–17.04)		8/81	9.88 (4.86–18.53)
Perth and Kinross, Scotland	2/33	6.06 (0.68–20.60)		10/33	30.30 (17.25–47.46)
Dorset, England	2/72	2.78 (0.19–10.15)		0/70	0.00 (0.00–6.23)
Cumbria, England	2/95	2.11 (0.12–7.81)		4/95	4.21 (1.31–10.67)
Argyll and Bute, Scotland	3/158	1.90 (0.40–5.69)		5/158	3.16 (1.16–7.39)
Wiltshire, England	1/56	1.79 (0.00–10.34)		5/55	9.09 (3.53–19.99)
Stirling, Scotland	0/2	0.00 (0.00–70.98)		1/2	50.00 (9.45–90.55)
Somerset, England	0/13	0.00 (0.00–26.59)		1/13	7.69 (0.00–35.42)
Moray, Scotland	0/19	0.00 (0.00–19.79)		1/19	5.26 (0.00–26.48)
Gloucestershire, England	0/24	0.00 (0.0016.31)		1/24	4.17 (0.00–21.87)
Aberdeenshire, Scotland	0/32	0.00 (0.00–12.73)		1/32	3.13 (0.00–17.11)

*HAI, hemagglutination inhibition. †95% CIs computed by Agresti Coull method.

Of all ticks submitted from deer carcasses, 2,041 collected from 339 deer were from within 15 km of an ELISA-positive result. All ticks were identified as *I. ricinus*; 1,450 were adult females, 585 adult males, and 6 nymphs. Tick availability for testing by area of seropositive foci varied (Figure 1, panel C); most ticks tested were collected from Argyll and Bute, and an average of 6 ticks were tested per deer. Five (4 adult males, 1 adult female) of the 2,041 ticks tested positive by the LIV/TBEV rRT-PCR (*20*) and were all within the Norfolk/Suffolk focus (Figure 1, panel C). No LIV RNA was detected in these 5 ticks when they were tested by rRT-PCR designed to detect only LIV (*21*). The 192 ticks tested from within the Norfolk/Suffolk focus resulted in a prevalence of 2.6% in this area.

**Figure 2:**
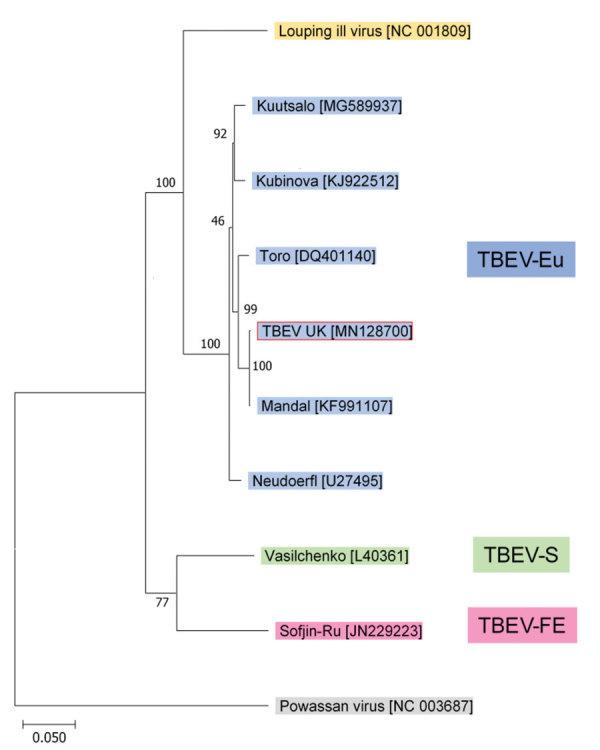
Phylogenetic relationship between TBEV-UK from a tick in the United Kingdom and contemporary strains of TBEV. The tree was constructed with a maximum-likelihood analysis using full-length complete TBEV genomes and is rooted with the tickborne Powassan virus. GenBank accession numbers of each sequence are provided in brackets. TBEV, tick-borne encephalitis virus; TBEV-Eu, TBEV-European; TBEV-FE, TBEV-Far Eastern; TBEV-S, TBEV-Siberian; TBEV-UK, TBEV-United Kingdom.

One tick (male) showed high levels of TBEV RNA (cycle threshold 15.4). Sequencing revealed a full-length TBEV genome designated TBEV-UK (GenBank accession no. MN128700). Phylogenetic analysis illustrates this as a TBEV-Eu subtype; it is most closely related to the Norwegian Mandal strain of TBEV isolated from ticks in 2009 (Figure 2), sharing a 99% sequence identity.

## Discussion

The detection of TBEV in the United Kingdom is important because TBEV can infect humans, causing febrile illness and neurologic complications including encephalitis. This evidence is contrary to earlier predictions based on climate change (*9*) that did not forecast a spread of TBEV to the United Kingdom. However, in addition to climate change, the spread of the tick vector, TBEV, and associated viruses into new regions can be influenced by a variety of other factors, such as transportation of animals and alterations in land management (*27*).

Serologic evidence suggests a high prevalence (47.7%) of exposure of deer to flaviviruses, such as TBEV and LIV, in the Norfolk/Suffolk (Thetford Forest) focal area. This seroprevalence is within the upper levels detected in TBEV risk areas of Europe, where seroprevalence studies in deer rarely exceed 50% (*15*,*16*,*28*,*29*). In addition, the detected prevalence of flavivirus RNA in ticks collected from deer of 2.6% within the Thetford Forest area falls within the range of findings from other studies in mainland Europe that tested blood-fed ticks (*15*). The deer were culled within a large forest habitat, which aligns closer with ecology required for TBEV, rather than LIV, maintenance (*7*). Based on these findings, and the evidence that all rRT-PCR–positive results were for TBEV and not LIV, we propose that TBEV is established and is being maintained through enzootic cycles within the Thetford Forest area, rather than resulting from multiple importation events, which is in line with findings in many endemic focal areas of TBEV (*30*). The hypothesis that TBEV infection might be maintained in Thetford Forest is supported by previous work in the United Kingdom that provided evidence of co-feeding between ticks from different life stages on small mammals in a southern English woodland, which is a crucial factor for the maintenance of TBEV (*9*,*12*). In addition, our positive serology data support the concept that the virus is circulating nonviremically in local wildlife and by cycling among the co-feeding nymphs, larvae, and adult ticks, through nonviremic transmission. Good evidence shows that TBEV is maintained in other parts of Europe through nonviremic transmission (*31*).

In other study areas, we detected serologic evidence of flavivirus exposure but not viral RNA in ticks. The close homology between LIV and TBEV presents challenges when serologic methods are used alone because the tests cannot distinguish between them. Thus, based on such data, confirming which virus is responsible for the seroreactivity in the areas where LIV has previously been reported is not possible. Previous reports of LIV prevalence are limited; just one study showed up to 15.3% of ticks positive for LIV (*32*). However, other researchers have not confirmed these data, and our results indicate a much lower prevalence. Nevertheless, we did not find any published clinical reports of LIV in Hampshire livestock despite our detected seroprevalence of 14.3% by ELISA (*1*,*3*,*4*). Although additional tick and small mammal ecology studies are needed to build on serologic data, evidence shows the maintenance of TBEV in the identified focal endemic area.

The genomic sequence of TBEV-UK shows close identity to a TBEV-Eu virus isolated in 2009 from questing ticks collected in Norway. This similarity suggests that TBEV-UK might have been brought to the United Kingdom on migratory birds, such as blackbirds (*Turdus merula*) and redwings (*Turdus iliacus*) (*33*), which are known to transport ticks over wide distances (*34*–*37*). The United Kingdom experiences a large influx of migratory birds each autumn from several TBEV-endemic countries in northern Europe, including Norway. During this migration, birds first arrive on the east coast of the United Kingdom, and it is feasible that TBEV-UK could have originated from a tick imported by an autumn migratory bird. We are collecting tick samples from migratory birds to assess the proportion of tick-infested birds arriving in the United Kingdom and testing these imported ticks for TBEV (among other potential pathogens). In addition, because of lower viral RNA levels, we are looking into primer-amplification sequencing approaches to further decipher the virus responsible for the rRT-PCR–positive samples detected from Thetford Forest.

For zoonotic infections, detection of a pathogen in the animal reservoir/host, vector, or both often precedes the emergence of human infection (*38*). Such was the case in the Netherlands, where deer serum samples, collected 6 years before the first cases in humans (*13*), demonstrated serologic evidence of TBEV infection (*14*). Similarly, in Spain, Crimean-Congo hemorrhagic fever virus was first reported in ticks in 2010 (*39*), before autochthonous infections in humans was identified in 2017 (*40*). Within the focal TBEV-endemic areas we identified in this study, seroepidemiologic studies should be undertaken, particularly in risk groups that include patients presenting to general practitioners and hospitals with central nervous system symptoms. 

Although UK-TBEV has not been linked to hu­man disease, it nevertheless shows close homology to pathogenic isolates of TBEV and should be considered to be a potential public health risk. Thus, clinicians in the United Kingdom should consider the European Union case definition of TBE (*41*) and include TBE in the differential diagnosis of patients with symptoms of meningoencephalitis, especially if they have been exposed to a tick bite, even if they have not traveled recently to a known TBE-endemic country. The European Union case definition specifies clinical criteria as any person presenting with inflammation of the central nervous system. In addition to meeting clinical criteria, laboratory case confirmation requires >1 of the following 5 criteria to be satisfied: 1) detection of TBEV nucleic acid, 2) viral isolation from clinical specimens, 3) TBEV-specific IgM and IgG in blood, 4) TBEV-specific IgM in cerebrospinal fluid, and 5) seroconversion or 4-fold increase of TBEV-specific antibodies in paired serum samples (*41*).

Although no autochthonous cases of clinical human disease have been diagnosed in the United Kingdom, up to 60% of encephalitis cases reach no diagnosis (*42*). Therefore, our results indicate that TBEV should be considered as a potential cause in encephalitis patients, and the wide distribution of the natural vector in the United Kingdom indicates a need for close monitoring and a potential for geographic spread and expanding risk areas.
